# Comprehensive evaluation of breast cancer immunotherapy and tumor microenvironment characterization based on interleukin genes-related risk model

**DOI:** 10.1038/s41598-022-25059-8

**Published:** 2022-11-28

**Authors:** Yalei Lv, Zihe Bai, Xiaoyan Wang, Jiayin Liu, Yuntao Li, Xiaolin Zhang, Yujie Shan

**Affiliations:** 1 Department of Medical Oncology, Fourth Hospital of Hebei Medical University, 12 Jiankang Road, Shijiazhuang, 050011 China; 2 The Fifth Ward of Medical Oncology, Shijiazhuang People’s Hospital, Shijiazhuang, China; 3 Breast Center, Fourth Hospital of Hebei Medical University, Shijiazhuang, China; 4 Department of Epidemiology and Statistics, Hebei Medical University, Shijiazhuang, China

**Keywords:** Cancer, Biomarkers, Oncology

## Abstract

Breast cancer (BRCA) is the most prevalent malignancy and the leading cause of death in women. Interleukin (IL) genes are critical in tumor initiation and control. Nevertheless, the prognosis value of the IL in BRCA remains unclear. We collected data from The Cancer Genome Atlas (TCGA) and Gene Expression Omnibus (GEO), and 94 IL genes were identified from GeneCard. Based on the random forest (RF), least absolute shrinkage and selection operator (LASSO) analysis, and multivariate Cox regression analysis, we constructed an IL signature. GSE22219, GSE25065, and GSE21653 were derived as validation sets. The expression differences in the tumor microenvironment (TME), immunotherapy, and chemosensitivity of BRCA between the high- and low-risk groups were evaluated. Overall, 21 IL genes were selected to construct an IL risk model, of which IL18BP, IL17D, and IL23A were the first time identified as prognostic genes in BRCA. IL score could distinguish BRCA patients with inferior outcomes, and AUC of it was 0.70, 0.76, and 0.72 for 1-,3- and 5- years, respectively, which was also verified in GSE22219, GSE25065, and GSE21653 cohorts. Meanwhile, compared to luminal A and luminal B, HER2-positive and TNBC had significantly higher IL score. Besides, the high-risk group had a significantly higher prevalence of *TP53* and *TTN* but a lower prevalence of *PIK3CA*, as well as higher tumor mutation burden (TMB) and neoantigen level. High- and low-risk groups exhibited notable differences in immunomodulators and tumor infiltrates immune cells (TIICs), and the high-risk group had significantly lower Tumor Immune Dysfunction and Exclusion (TIDE) score. Additionally, the high-risk group has more responders to immune or anti-HER2 combination therapy, whereas the low-risk group has higher sensitivity to docetaxel and paclitaxel. Consequently, we constructed a reliable risk model based on the IL genes, which can provide more information on both the risk stratification and personalizing management strategies for BRCA.

## Introduction

Breast cancer (BRCA) is the most frequently diagnosed malignancy and the fifth greatest cause of cancer-related death in women worldwide. Despite revolutionary advances have been made in the early-detection, there are still approximately 2.3 million new cases diagnosed with it in 2020, accounting for 11.7% of all cancer cases worldwide^1^. Meantime, BRCA is a widely known heterogeneous disease that can be classified into different histopathological subtypes according to the expression of hormone receptors (estrogen receptor (ER) and progesterone receptor (PR)) and epidermal growth factor receptor 2 (HER2/Neu)^2,3^. Then, different treatment methods, such as surgery, radiation therapy, chemotherapy, and endocrine would be adopted according to the patient’s histological subtype^4^. However, primary and or developed subclones promoted by therapeutic drugs would further result in drug resistance and tumor recurrence^5^. In addition, primary defined histological subtypes, such as triple-negative breast cancer (TNBC), are also molecularly heterogeneous and can be further categorized into subtypes with varying prognoses^6^. Mounting evidence demonstrates the emerging role of immunotherapy in the BRCA treatment, and emphasizes the association between tumor microenvironment and BRCA metastasis^7,8^. Lv et al.^9^ generated an aging genes-related risk stratification that can be used for predicting immunotherapy in BRCA. Zhou et al.^10^ developed a prognostic model of cellular senescence-related genes, which was applied to characterize the tumor microenvironment infiltration in BRCA. Nevertheless, the existing risk models still have an inadequacy in the prognostic role, thus, it is of great significance to construct a novel signature for improving the prognosis of BRCA.

Interleukins (IL) and associated cytokines regulate the innate and adaptive immunity in normal and tumor tissue, with roles in immunomodulatory, promoting signal transduction, and maintaining tissue homeostasis^11,12^. Previous studies have indicated that IL servers as a “double agent” in tumor development: on one hand, chronic inflammation has been recognized as a driver of carcinogenesis which produces carcinogenic mediators, and IL further promotes tumor growth, metastasis and progression^13^; on the other hand, IL also governs the innate and adaptive immunity-mediated cancer cell death, starting from the lymphocytes’ proliferation to the termination^13,14^. Specifically, numerous studies have delineated the role of IL in the development of BRCA, and most of them have long been implicated as the promoters contributed to tumor invasion, migration and therapy resistance^15–17^. For instance, IL6 not only interacts with STAT3 signaling to drive ER positive BRCA metastasis and lead to resistance to hormone therapy^18^, but also alters tumor microenvironment (TME) in TNBC promoting epithelial-mesenchymal transition (EMT) progression, cancer stemness and M2 macrophage polarization^19^. On the contrary, some IL family members have also been proven with antitumor activity in BRCA^20,21^. Overexpression of IL-1β has been revealed the association with better outcomes in BRCA patients with lymph node metastasis^22^. Moreover, as IL directly participates in or mediates the regulation of the TME, it could be targeted to increase the sensitivity of immune checkpoint inhibitors^23^. Prior research mainly has only established a correlation between a single IL gene and BRCA prognosis^24–26^; hence, the comprehensive prognostic function of IL family genes in breast cancer, to our best knowledge, remains unknown.

In this research, the underlying role of IL in prognosis, gene mutation, TME, and immunotherapy of BRCA were our primary concerns. Based on the IL genes, we have developed an IL score that could predict the OS of BRCA patients in the TCGA cohort and further validated this risk model in three GEO cohorts. In addition, the correlation of mutation characteristics and risk score were comprehensively applied, and we further evaluated the TME landscape between high- and low-risk groups. Moreover, the response to immunotherapy in BRCA patients with different IL scores was performed to contribute reliable insights into the treatment for BRCA. Taken together, our study might provide robust biological targets for improving prognosis and clinical treatment for BRCA patients, and offer further reliable guidance on the personalized medicine of BRCA.

## Materials and methods

### Data collection and analysis

The RNA expression data and clinical details of the TCGA-BRCA cohort were downloaded from The Cancer Genome Atlas (TCGA) database (http://xena.ucsc.edu/) as the training set, with male BRCA patients excluded. Meanwhile, the GSE22219, GSE25065, and GSE21653 were collected from Gene Expression Omnibus (GEO) (https://www.ncbi.nlm.nih.gov/geo/) and were used as three independent validation sets. Meantime, we searched “interleukin” as a keyword from the GeneCard (https://www.genecards.org/) with the category protein-coding and high correlation score, and finally a total of 94 IL genes (Table S1) were identified and utilized for the subsequent analysis.

### Construction and validation of the IL score

Firstly, we performed random forest (RF) with R package “randomSurvivalForest” (version 3.6.4)^27^ to obtain hub IL genes (with variable relative importance ≥ 0.4). Secondly, the least absolute shrinkage and selection operator (LASSO) regression analysis^28^ was applied to screen out the optimal genes through the "glmnet" R package^29^, and the best λ value was obtained by tenfold cross-validation used for gene screening. We took the intersection genes of hub genes and optimal genes from RF and LASSO, respectively, and applied multivariate Cox regression analysis to construct a risk model based on them. The risk model formula was exhibited as follows: IL score = ${\sum }_{j=1}^{n}{Expr}_{genej}*{Coef}_{genej}$, and the BRCA patients were divided into a high-risk group and a low-risk group according to the median IL score. To further evaluated the prognostic ability of the IL score, we used the Kaplan–Meier survival curve to evaluate the overall survival (OS) ability based on the “survival" and “survminer” packages^30^, and performed receiver operating characteristic (ROC) at 1, 3 and 5 years to calculate the area under the curve (AUC) values using the “timeROC” R package.

### Clinical features analysis and nomogram establishment

The Kruskal Wallis test was used to analyze the difference in clinical characteristics between two risk groups, including age, tumor node metastasis (TNM) and pathological stage, and the stratified analysis was then performed in the TCGA cohort. According to the results of univariate and multivariate Cox regression analysis, we generated a nomogram based on IL score and clinicopathological parameters using “rms” R package, and the performance of the nomogram was assessed by calibration curves and ROC curves.

### Molecular subtypes of BRCA

BRCA was mainly divided into four molecular subtypes, including luminal A, luminal B, HER2-positive, and triple-negative BRCA (TNBC)^31^. Then, we performed an alluvial diagram to visualize the changes in IL score in four molecular subtypes of BRCA and applied the Kaplan–Meier survival curves to analyze the prediction ability in the IL score of four molecular subtypes with “survival” and “survminer” packages. In addition, we compared the differences in IL score, tumor mutation burden (TMB), and neoantigen level among four different molecular subtypes of BRCA.

### Functional enrichment analysis

To deeply explore the underlying biological activities of the risk model, we firstly screen the genes that were strongly associated with IL score using the Pearson (|R|> 0.25, p < 0.05), and we finally obtained IL score -associated genes. Kyoto Encyclopedia of Genes and Genomes (KEGG)^32^ and Gene Ontology (GO)^33^ functional annotation was applied to analyze the functional enrichment of IL score-associated genes using the “cluster Profile” R package. The GO mainly performed enrichment analysis in three aspects, including biological process (BP), cellular component (CC), and molecular function (MF). p-value < 0.05 and q < 0.05 were set as cutoff values.

### Mutation analysis

To evaluate the mutational landscape of BRCA patients between high- and low-risk groups, we analyzed the mutation information of BRCA patients using “maftools” package. Subsequently, we used cBioPortal (https://www.cbioportal.org/mutation_mapper) to draw lollipop plots that could identify the variation distribution of genes and the underlying functional impact of mutations. Finally, the TMB of the high- and low-risk groups was also assessed.

### Tumor microenvironment of BRCA

The tumor microenvironment (TME) included non-cancerous host cells in the tumor and non-cellular components, which played an important role in the progression and therapeutic effect of cancer^34^. To identify the immunological features of the TME in BRCA, we assessed the expression level of immunomodulators between high- and low-risk groups. In addition, we performed CIBERSORT^35^ to analyze tumor infiltrates immune cells (TIICs) abundance in high- and low-groups.

### The immunotherapy landscape

First of all, we evaluated the different expression levels between 24 immune checkpoints and two risk groups. Moreover, we calculated the enrichment scores of immunotherapy prediction pathway between high- and low-risk groups in the TCGA cohort, and the ESTIMATE algorithm was applied to calculate the immune score, stromal score, and tumor purity. Tumor Immune Dysfunction and Exclusion (TIDE) score, T cell dysfunction score, T cell exclusion score, and microsatellite instability (MSI) score were evaluated based on the TIDE website (http://tide.dfci.harvard.edu/). In addition, the dataset GSE173839 was applied to evaluate the immunotherapy benefit of the risk model. Finally, we downloaded the immunophenoscore (IPS) of BRCA from The Cancer Immunome Atlas (TCIA) (https://tcia.at/home) to predict the BRCA patients’ response to immunotherapy.

### The correlation between chemosensitivity and IL score

We collected three treatment targets, including EGFR network, immune inhibited oncogenic pathways, and radiotherapy predicted pathways to assess the correlation between treatment targets and the risk model. In addition, the drugbank database (https://go.drugbank.com/) was used to obtain the BRCA-related drug target genes. Furthermore, we used the “ggplot2” R package to compare the proportion of pathological complete response (pCR) among patients in the high- and low-risk groups who received neoadjuvant chemotherapy in the GSE194040 cohort.

### Statistical analysis

We used R software and its associated software package (v.4.1.2) to analyze the data. Kaplan–Meier curves and log-rank tests were used to assess differences in OS between groups. Continuous data processing was performed by Student's t-test and Wilcoxon's test, and Fisher's exact test was applied to categorical data. All tests were two-way and p < 0.05 was considered statistically significant.

## Results

### Development of the IL score

**Figure 1:**
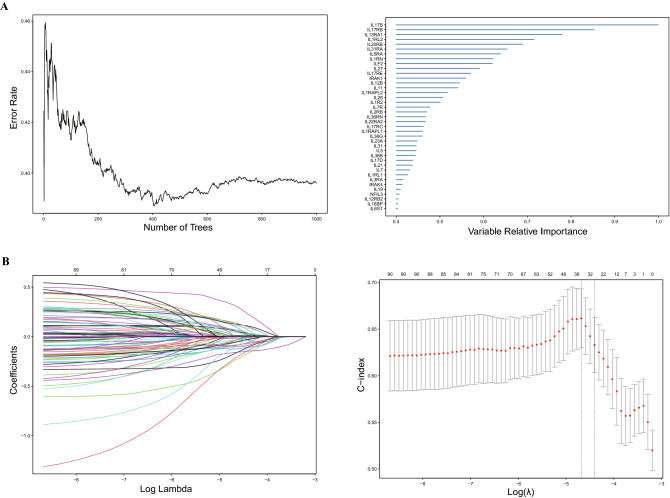

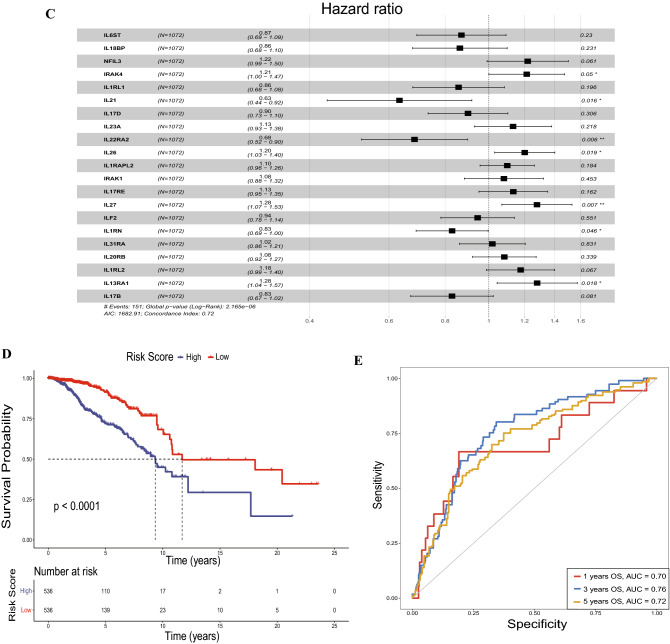
Construction of IL score. (**A**) Screening hub genes by RF with variable relative importance ≥ 0.4. (**B**) Screening optimal genes by LASSO and calculating the minimum criteria. (**C**) Multivariate Cox regression analysis to obtain intersection genes. (**D**) Kaplan–Meier curve in TCGA cohort. (**E**) Sensitivity and specificity of ROC curve in TCGA cohort. IL, interleukin; RF, random forests; LASSO, Least Absolute Shrinkage and Selection Operator; TCGA, The Cancer Genome Atlas; ROC, receiver operating characteristic; AUC, area under the curve.

First, we used RF to screen the IL genes and found 39 hub IL genes with relative significance values greater than 0.4 (Fig. 1A). In the meantime, LASSO regression analysis further determined 42 optimal IL genes (Fig. 1B) (Table S2). Then, 21 intersection genes were overlapped by these two algorithms, including IL6ST, IL18BP, NFIL3, IRAK4, IL1RL1, IL21, IL17D, IL23A, IL22RA2, IL26, IL1RAPL2, IRAK1, IL17RE, IL27, ILF2, IL1RN, IL31RA, IL20RB, IL1RL2, IL13RA1, and IL17B (Table S3). Based on the 21 IL genes, the multivariate Cox regression analysis was employed, aiming to construct a risk model (Fig. 1C). The IL score was calculated by multiplying the expression of each gene by its respective coefficient and adding the results together (Table S4). The BRCA patients in the training cohort were divided into high- and low-risk groups according to the median IL score. Figure S1 displayed the distribution of risk score and survival status in BRCA patients. Low-risk patients had better OS than those with high-risk (median OS: 9.3 months vs 11.7 months, p < 0.0001, Fig. 1D), and the AUC for prediction survival of the IL score was 0.70 in 1 year, 0.76 in 3 years, and 0.72 in 5 years (Fig. 1E).

### Validation and comparison of IL score

**Figure 2:**
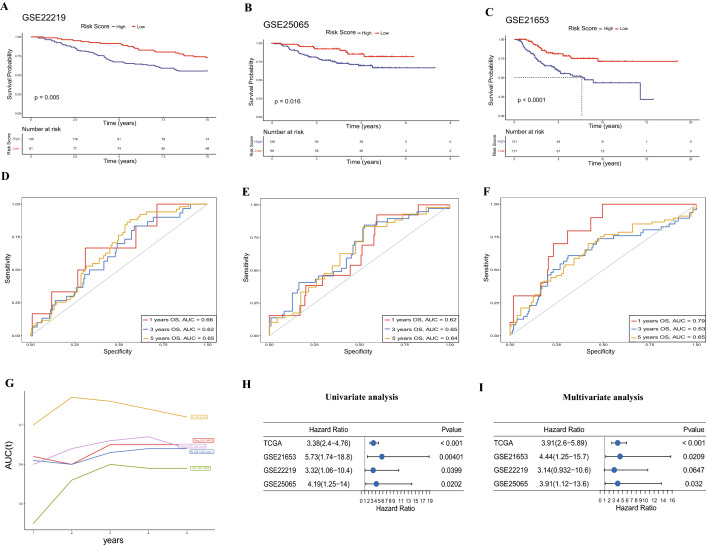
Validation of IL score. Kaplan–Meier curves in validation cohorts of GSE22219 (**A**), GSE25065 (**B**), and GSE21653 (**C**). Sensitivity and specificity of ROC curves in validation cohorts of GSE22219 (**D**), GSE25065 (**E**), and GSE21653 (**F**). (**G**) Comparison of ROC curves for five risk models. Forest plots of IL score in TCGA, GSE21653, GSE22219, and GSE25065 by univariate Cox regression analysis (**H**) and multivariate Cox regression analysis (**I**).

To further access the robustness of the IL score, we performed three independent cohorts from GEO, including GSE22219, GSE25065, and GSE21653 using the same formula to calculate the risk score (Table S5–S7). Figure S2A–C showed the distribution of risk score and survival status for BRCA patients in three validation sets. As shown in the Fig. 2A–C, high-risk patients in three validation cohorts all had worse OS than low-risk patients. In addition, the AUC values of 1-, 3-, 5 years were 0.66, 0.62 and 0.65 in GSE22219 cohort (Fig. 2D); 0.62, 0.65 and 0.64 in GSE25065 cohort (Fig. 2E); and 0.79, 0.63 and 0.65 in GSE21653 cohort (Fig. 2F). To comprehensively analyze the prognostic ability of IL score, four risk models^36–39^ from previous studies were collected to compare with the prognostic prediction capacity. The result demonstrated that the AUC value of the IL score was significantly greater than the other four risk models, showing that our risk model surpassed the others in terms of accuracy (Fig. 2G). In addition, both univariate and multivariate Cox regression analyses demonstrated that the IL score had good prognostic value in the four cohorts (Fig. 2H and I).

### The landscape of clinicopathological features in the risk model

**Figure 3:**
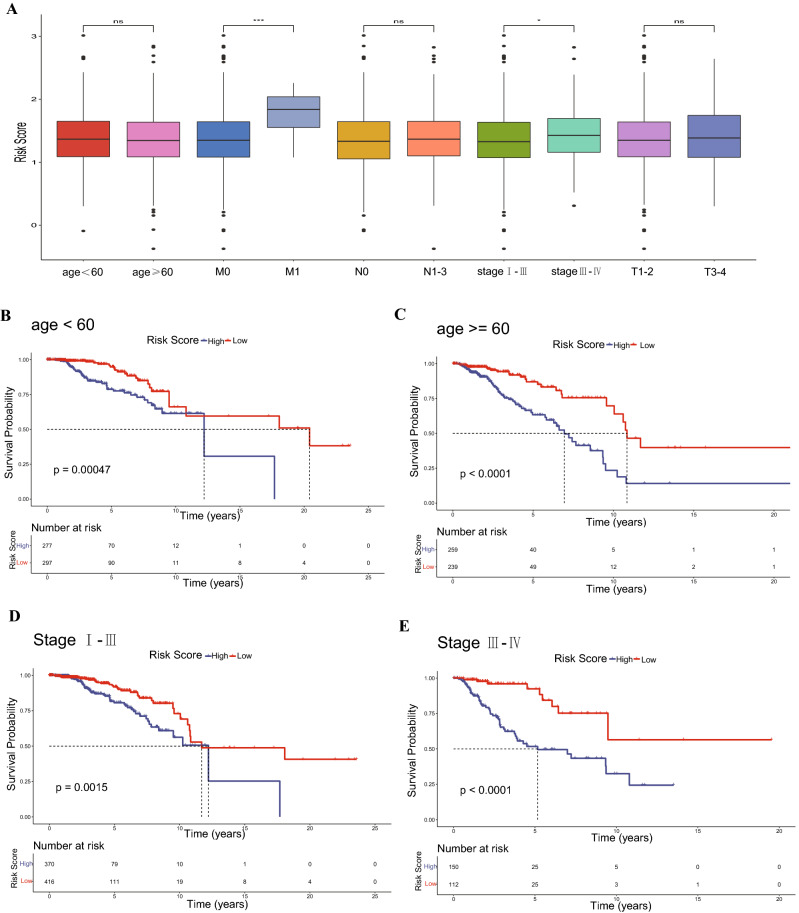

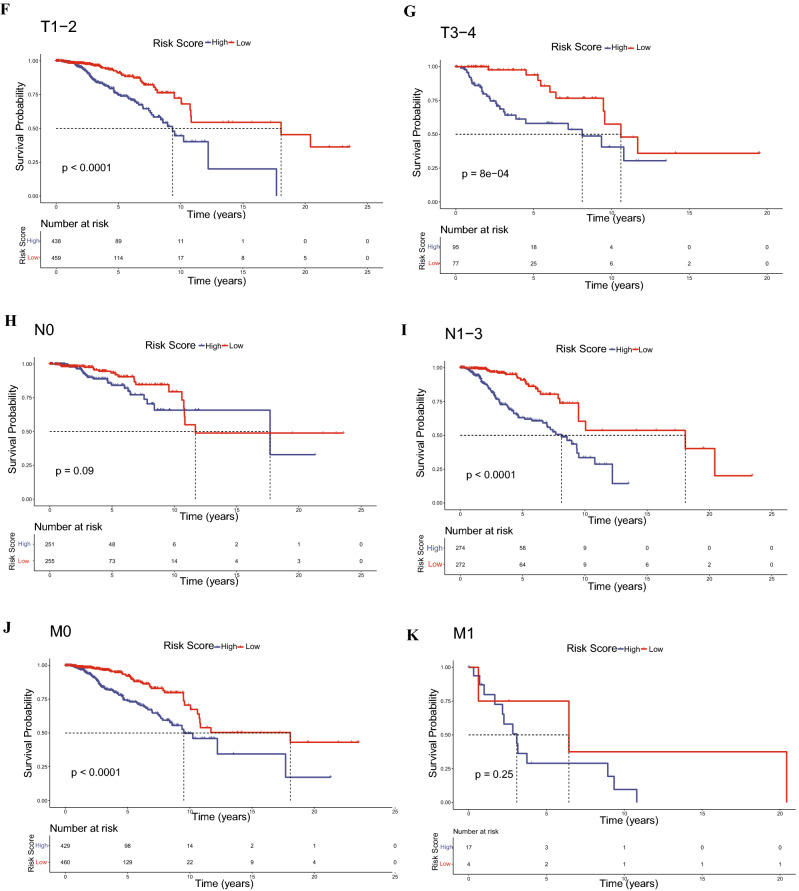
The association between clinical characteristics and IL score. (**A**) Difference between clinical features and IL score. (**B**) Survival analysis of clinical features, including age < 60 years (**B**), age ≥ 60 years (**C**), stage I–II (**D**), stage III–IV (**E**), T1–2 (**F**), T3–4 (**G**), N0 (**H**), N1–3 (**I**), M0 (**J**) and M1 (**K**).

Then, we analyzed the difference in IL score across BRCA patients with different clinicopathological features, including age, pathological stage, and TNM stage, and found that patients with M1 or higher stages had a significantly higher risk score (Fig. 3A). In addition, stratification survival analysis was applied according to age (< 60 years and ≥ 60 years), pathological stage (stage I–II and stage III–IV), T stage (T1–2 and T3–4), N stage (N0 and N1–3), and M stage (M0 and M1). It was confirmed that the patients with high risk score had significantly shorter OS, regardless of their clinicopathological features (Fig. 3B–K).

### Heterogeneity in IL score between different molecular subtypes

**Figure 4:**
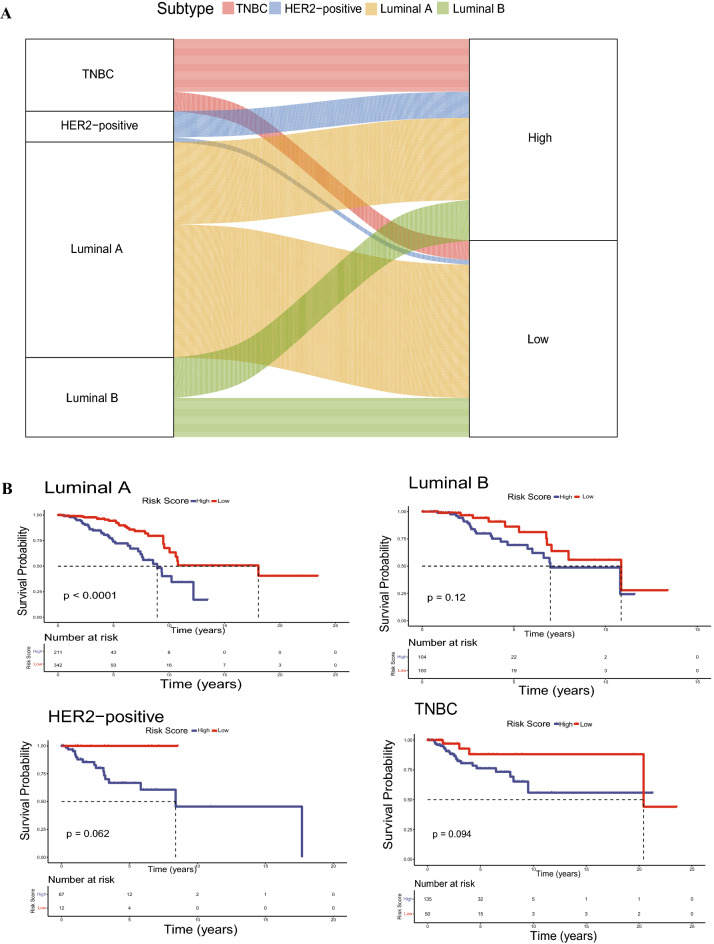

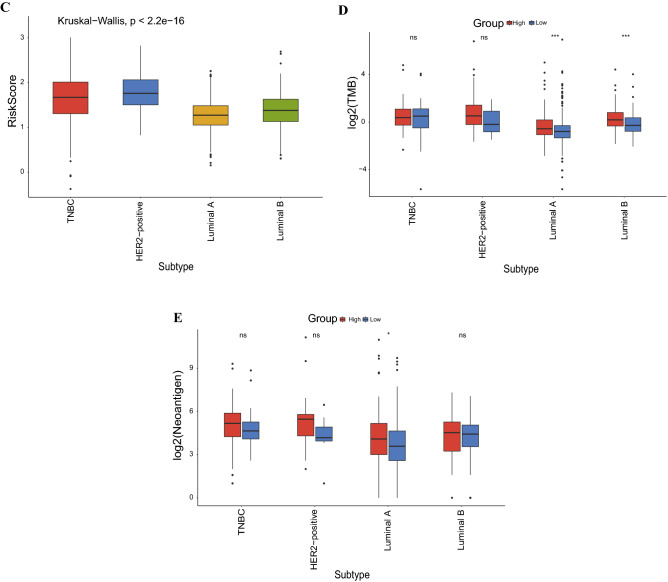
Molecular subtypes in BRCA. (**A**) Alluvial diagram of four molecular subtypes and IL score. (**B**) Kaplan–Meier curves of four molecular subtypes. (**C**) Differences in IL scores among the four molecular subtypes. (**D**) TMB in the four molecular subtypes. (**E**) Neoantigen levels of four molecular subtypes. BRCA, breast cancer; TMB, tumor mutation burden.

The interaction between well known four molecular subtypes (luminal A, luminal B, HER2-positive, and TNBC) and IL score subtypes were visualized using an alluvial diagram (Fig. 4A). Intriguingly, there were more TNBC and HER2-positive patients in the high-risk group, whereas there were more luminal A patients in the low-risk group. Survival analysis revealed that the IL score may distinguish the survival of luminal A patients significantly (p < 0.0001). The similar trend was observed in the other three subtypes, although there was no statistically significant difference due to limited sample size (Fig. 4B). HER2-positive and TNBC had the highest IL score compared to the other molecular subtypes, which was consistent with aforementioned result in Fig. 4A (p < 2.2e−16, Fig. 4C). Moreover, TMB values were both significantly elevated in the high-risk group of luminal A and luminal B patients (all p < 0.001, Fig. 4D). Subsequently, neoantigen levels were significantly higher in the high-risk group of luminal A patients (p < 0.05, Fig. 4E).

### Nomogram construction

**Figure 5:**
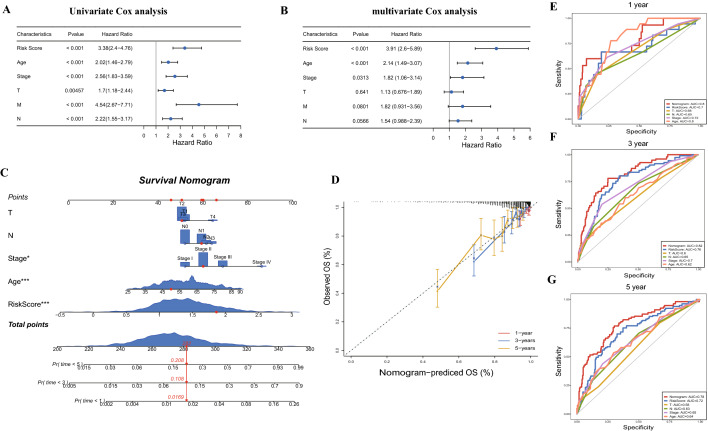
Construction of nomogram. (**A**) Univariate Cox regression analysis. (**B**) Multivariate Cox regression analysis. (**C**) Nomogram for predicting the probability of 1-, 3-, and 5-years OS in the TCGA cohort. (**D**) Calibration curves for predicting the fitness of the nomogram in 1-, 3-, and 5-years. ROC curves of a nomogram for 1- (**E**), 3- (**F**), and 5-year (**G**) in the TCGA cohort. OS: overall survival.

Univariate and multivariate Cox regression analysis revealed that the IL score was an independent prognostic indicator (HR = 3.91, 95% CI 2.60–5.89, p < 0.001, Fig. 5A and B). Next, we constructed a nomogram according to IL score, T stage, N stage, pathological stage, and age (Fig. 5C), which showed a good agreement between actual and predicted survival at 1-, 3- and 5-year (Fig. 5D). The AUC for prediction survival at 1-, 3- and 5- year was further improved to 0.80, 0.82 and 0.78, respectively, outperforming the risk score and other clinicopathological factors (Fig. 5E–G).

### Functional enrichment analysis

**Figure 6:**
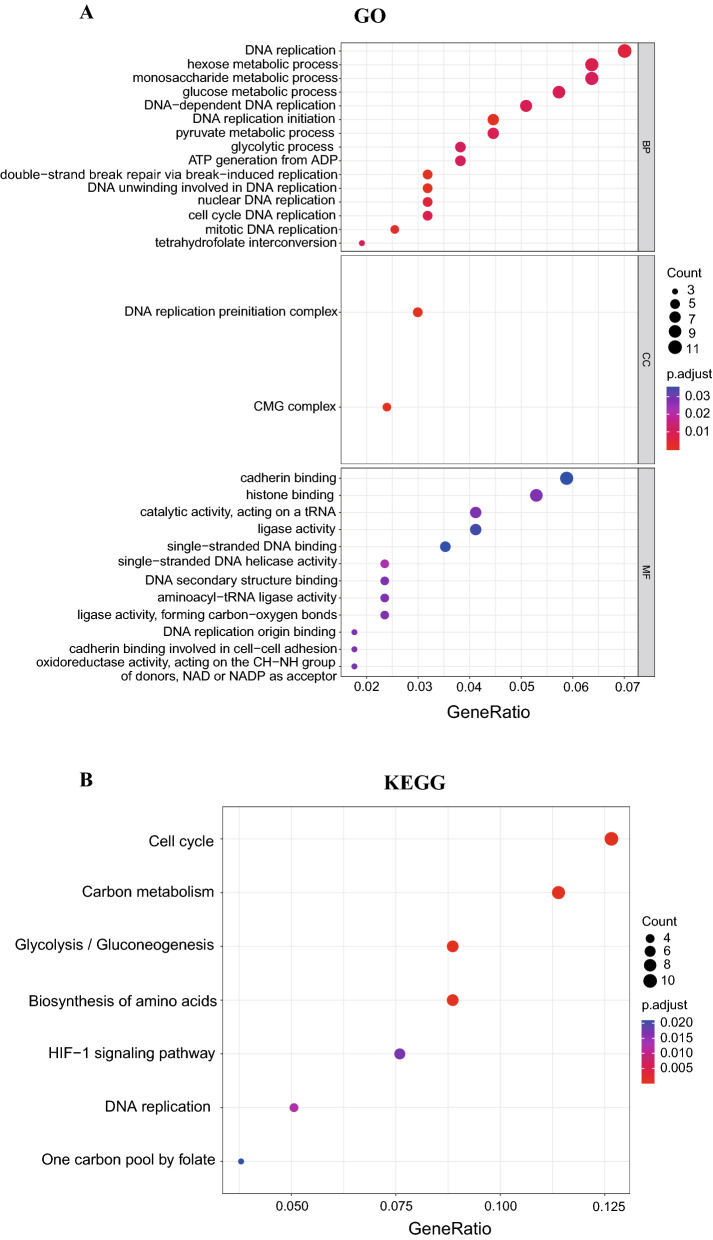
Pathway and enrichment analysis of IL score-associated genes. (**A**) GO analysis. (**B**) KEGG analysis. GO, Gene Ontology; KEGG, Kyoto Encyclopedia of Genes and Genomes; BP, biological process; CC, cellular component; MF, molecular function.

To investigate the potential biological activities of the risk model, we performed the GO and KEGG enrichment analysis based on the IL score-associated genes (R-value > 0.25). The results revealed that the IL score-associated genes were primarily enriched in DNA replication, hexose metabolic process, and monosaccharide metabolic process. From the aspect of GO CC, we found these genes mainly clustered in the DNA replication preinitiation complex. And cadherin binding, histone binding, and catalytic activity acting on a tRNA were major concentrated in GO MF (Fig. 6A). Through KEGG analysis we found that the IL score-associated genes were mainly involved in the cell cycle, carbon metabolism, glycolysis, and gluconeogenesis (Fig. 6B).

### Genomic landscape

**Figure 7:**
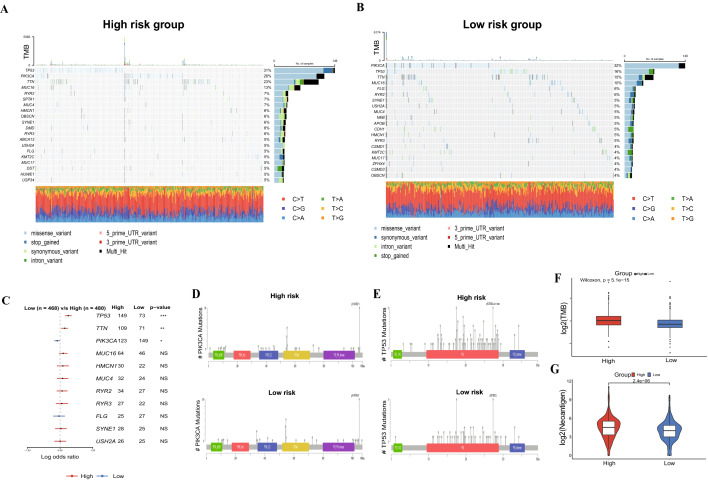
Genetic mutations analysis. Waterfall plots of somatic mutation features in the high-risk group (**A**), and low-risk group (**B**). (**C**) The forest plot exhibits the 11 genes with the highest differences between the high- and low-risk groups. (**D**) Lollipop plots showing *PIK3CA* gene mutations in the high- and low-risk groups. (**E**) Lollipop plots showing *TP53* gene mutations in the high- and low-risk groups. (**F**) The TMB between high-and low-risk groups. (**G**) The comparison of neoantigen levels between high- and low risk groups.

Somatic mutation profiles of BRCA patients from the TCGA cohort were visualized by waterfall plots, which showed that *TP53*, *PIK3CA*, and *TTN* were the most frequently mutated genes in both high-risk and low-risk groups (Fig. 7A and B). However, the prevalence of the top 3 genes, including *TP53*, *TTN*, and *PIK3CA* were significantly differed between the two groups according to Fisher’s test (all p < 0.05, Fig. 7C). In addition, as shown in lollipop plots, *PIK3CA* mutations were mainly distributed in specific functional domains, including PI3K-p85B, PI3K-C2, PI3Ka, and P13-P14-kinase (Fig. 7D); the distribution of *TP53* mutations was primarily in specific functional domain P53 (Fig. 7E). In addition, TMB was significantly higher in the high-risk group (p = 5.1e−15; Fig. 7F), which was accompanied by a rising level of neoantigen (p = 2.4e−06; Fig. 7G).

### Association between IL score and tumor microenvironment

**Figure 8:**
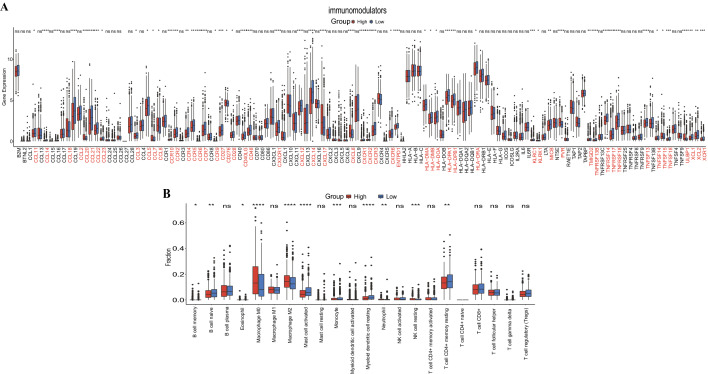
The role of IL score in TME. (**A**) Expression profile of immunomodulators in high-and low-risk groups. (**B**) The proportions of TIICs between high- and low-risk groups. TME, tumor microenvironment; TIICs, tumor infiltrates immune cells.

We explored the expression level of immunomodulators, and the distribution of these 122 immunomodulators in high- and low-risk groups was shown in Fig. 8A. Interestingly, though high risk group had notable elevated TMB value, more significantly overexpression of immunomodulators were presented in the low-risk group, including CCL1, CCL19, CX3CR1. Subsequently, we identified the differences in TIICs between the high- and low-risk groups, high-risk group had significantly higher abundances of macrophage M0, macrophage M2, neutrophil and NK cell resting, as well as lower abundances of B cell naïve, mast cell activated, monocyte, myeloid dendritic cell resting and T cell CD4+ memory resting than those of low-risk group (all p < 0.05, Fig. 8B).

### Correlation of the IL score with immunotherapy

**Figure 9:**
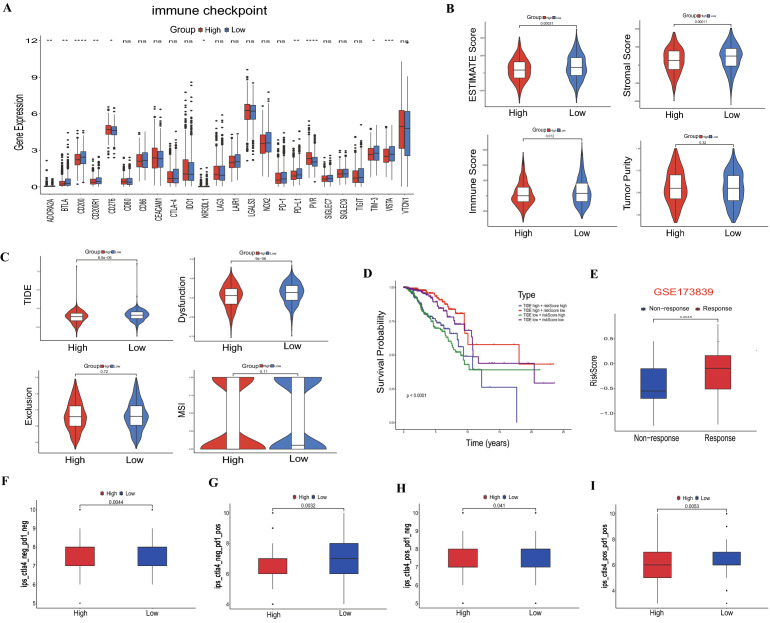
Immunotherapy landscape in BRCA. (**A**) The correlation between the immune checkpoints and IL score. (**B**) Relationship of IL score and ESTIMATE score, stromal score, immune score, and tumor purity. (**C**) Association of IL score and TIDE, T cell dysfunction, T cell exclusion, and MSI. (**D**) Survival analysis with different combinations of TIDE and IL scores. (**E**) The risk scores of non-response and response in the GSE173839 cohort. (**F**–**I**) IPS difference of BRCA with different statuses of CTLA-4 or PD-1. TIDE, Tumor Immune Dysfunction and Exclusion; MSI, microsatellite instability; IPS, immunophenoscore.

First of all, we analyzed the expression profile of 24 immune checkpoints between the high- and low-risk groups. The expression of PD-L1, ADORA2A, BTLA, CD200, CD200R1, KIR3DL1, TIM-3, and VISTA was more prevalent in the low-risk group, whereas CD276 and PVR were up-regulated in the high-risk group (Fig. 9A). We determined, using the ESTIMATE algorithm, that the ESTIMATE score, stromal score and immune score of the low-risk group were significantly higher than those of the high-risk group (Fig. 9B). In addition, the TIDE score (p = 6.5e−06) and T cell dysfunction score (p = 9e−06) were markedly lower in the high-risk group, indicating that the high-risk group may have a higher possibility to respond to immunotherapy (Fig. 9C). The survival analysis further showed that BRCA patients with high TIDE and lower risk score had the best OS (p < 0.0001, Fig. 9D). We subsequently applied GSE173839 to further explore the predictive ability of IL score for immunotherapy response in BRCA. Responders to durvalumab and olaparib therapy had higher risk score than non-responder in the GSE173839 (p = 0.0045, Fig. 9E). Additionally, anti-CTLA4 and anti-PD-1 therapy, either alone or in combination, were more beneficial for patients in the low-risk group (p = 0.0044; p = 0.0032; p = 0.041; p = 0.0053; Fig. 9F–I). In consequence, we believed that the risk model had the potential to be employed to identify the immunotherapy response of BRCA patients.

### Chemotherapeutic sensitivity and treatment efficiency of IL score

**Figure 10:**
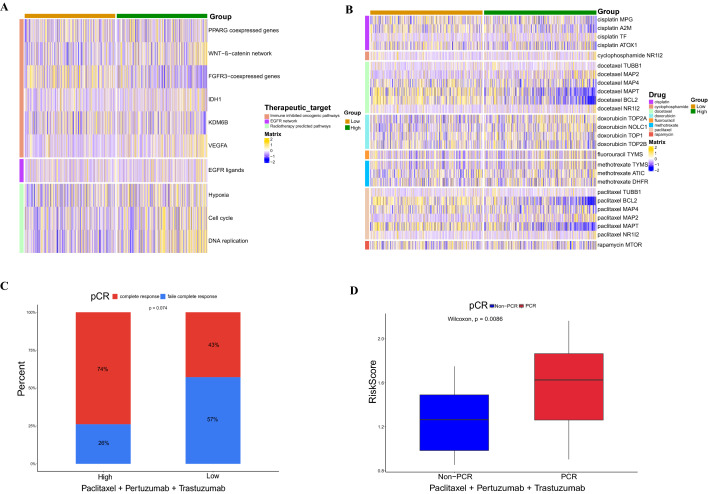
Sensitivity analysis of chemotherapy. (**A**) Expression profiles of three treatment characteristics between high-and low-risk groups. (**B**) Correlation between chemotherapeutic drug target genes and IL score in BRCA. (**C**) The pCR ratio of neoadjuvant chemotherapy (paclitaxel + pertuzumab + trastuzumab) in the high- and low-risk groups. (**D**) Comparison of risk scores for neoadjuvant chemotherapy (paclitaxel + pertuzumab + trastuzumab) in non-pCR and pCR. pCR, pathological complete response.

We explored the relationship between IL score and therapeutic signatures, such as EGFR network, immune inhibited oncogenic pathways, and radiotherapy predicted pathways, the results showed that the high-risk group showed a significant positive correlation in the radiotherapy predicted pathway (Fig. 10A). Furthermore, the target genes from chemotherapy drugs indicated that the low-risk group was positively associated with response to docetaxel and paclitaxel (Fig. 10B). According to the GSE194040 cohort, the pCR proportion of neoadjuvant chemotherapy (paclitaxel + pertuzumab + trastuzumab) in the high-risk group was significantly higher than that in the low-risk group, but it did not reach statistical significance (p = 0.074, Fig. 10C), and twelve other neoadjuvant chemotherapy treatments were presented in Fig. S3. Moreover, neoadjuvant chemotherapy (paclitaxel + pertuzumab + trastuzumab) had a higher risk score in pCR than non-pCR (p = 0.0086, Fig. 10D), risk scores for the other eleven neoadjuvant chemotherapy treatments were shown in Fig. S4. These results illustrated the potential therapeutic implications of the risk model for chemosensitivity in BRCA.

## Discussion

In this study, multiple machine learning algorithms, such as RF, LASSO, and multivariate Cox regression analysis, were used to extensively examine the role of IL genes and ultimately create a 21 IL gene-related prognostic model in BRCA. Not only did high-risk and low-risk BRCA individuals exhibit varied prognoses, but also distinct clinicopathologic characteristics, TME landscape, immunotherapy, and chemotherapy. Our research demonstrated the predictive relevance, biological significance, and therapeutic potential of IL genes in BRCA.

As formentioned, IL plays an important role in human disease and is closely related to the occurrence and development of cancer^40^, and multiple IL genes have been confirmed their function in the proliferation and growth of BRCA cells^41,42^. Considering the critical role of IL genes in cancer, it is necessary to comprehensively investigate their prognostic value in BRCA. Notably, in current study, we found 21 genes with significant prognostic predictive functions, and IL18BP, IL17D, and IL23A were identified for the first time as being significantly correlated with the prognosis of BRCA. At the same time, IRAK4, IL21, IL22RA2, IL26, IL27, IL1RN and IL13RA1 were significantly related to the OS of BRCA. A previous research discovered that variants in IRAK4 are highly associated with the prognosis of BRCA, which increase nearly fivefold risk of developing BRCA^43^. As a member of the IL-10 family of cytokines, IL26 participates in inflammatory signaling and is overexpressed in TNBC^44^; nevertheless, a previous study found that the introduction of recombinant IL21 could enhance the anticancer impact of trastuzumab in the treatment of metastatic HER2-positive patients and that IL21 expression is essential for CD8 T cells to optimal anti-HER2 antibody effectiveness^45^. Furthermore, it was reported that the mutant alleles of IL1RN are correlated with shorter OS in BRCA patients, mainly by altering IL1 receptor binding and resulting in the production of IL-1, which produces a proinflammatory status and enhances the aggressiveness of BRCA tumor^46^. In addition, several studies also have confirmed that IL13RA1 is strongly related to the survival of BRCA^37,47^. All of these data corroborated our conclusions and demonstrated the prognostic function of IL genes in BRCA. Additionally, the IL gene play a key role on the biological functions, recent work from Liubomirski et al.^48^ found that the interaction of Notch with IL-6 and the transcription factor STAT3 enhances pro-tumor functions in BRCA. There is evidence that inactivating mutations of P53 can guide the loss of methylation of IL-6, leading to epigenetic reprogramming and driving the development of BRCA^49^. Accordingly, we constructed a 21 IL gene-related risk model in BRCA, which is proved to be robust and reliable with the AUC for OS of the IL score was 0.70 in 1 year, 0.76 in 3 years, and 0.72 in 5 years in the TCGA dataset. Besides, baesd on the nomogram, the AUCs for the OS predictions for 1, 3, and 5 years were 0.80, 0.82 and 0.78, respectively, demonstrating the practical application value of this risk model in clinical management for BRCA patients.

Moreover, the GO and KEGG enrichment analysis found that the DNA replication, glucose metabolic process, and glycolysis pathways were the most enriched in the pathways related to IL score. Studies have discovered that cancer is correlated with errors that occur during DNA replication^50^, and the inhibition of DNA replication is shown to be beneficial to improve the prognosis of BRCA^51^. Recent research has demonstrated the crucial role played by glucose metabolic process in the growth of TNBC^52^. Besides, glycolysis exhibits elevated activity in BRCA, mainly through the effect of mTOR hyperactivation^53^.

Furthermore, we systematically studied the genomic differences between high- and low-risk groups, and *TP53* was the most frequently mutated gene in the high-risk group. It has been reported that nearly one third BRCAs have *TP53* mutations, which reduces the transcriptional activity of p53, resulting in the development of BRCA^54^. Funda et al.^55^ have proved that BRCA patients with TP53 alterations had significantly inferior recurrence-free survival, progression-free survival, and overall survival. Accordingly, *TP53* mutations may contrbute to the poor prognosis in the high-risk group. On the contrary, the mutation frequency of *PIK3CA*, which is a prevalent gene in BRCA^56^, was higher in the low-risk group, and is also an effective prognostic marker of BRCA^57^. The increasing prevalence of PIK3CA may be explained by higher proportion of hormone receptor positive patients in the low risk group. As alpelisib, a α-selective PIK3CA inhibitor, has been approved by FDA for treating BRCA patients with this kind of mutants, low risk group had potentially higher clinical benefits to the targeted therapy^58^. Moreover, TMB is assoicated with a higher probability of response to immune checkpoint inhibitors (ICIs)^59^, and we found that high-risk group had a significantly higher TMB. As Targeted Agent and Profiling Utilization Registry (TAPUR) and GeparNuevo Study both revealed that ICI monotherapy showed satisfied antitumor activity in BRCA patients with high TMB^60,61^, it suggested that high risk patients may have higher respondse rate to ICIs.

Notably, more TNBC and HER2-positive BRCA patients were in the high-risk group, which was consistent with the fact that TNBC and HER2-positive BRCA patients exhibit highly invasiveness and high proliferation characteristics and have a worse prognosis than other types of BRCA^4,62^.This difference in subtype distribution was consistent with the finding in the TMB that TNBC had the highest TMB compared to other BRCA subtypes^63^. On the other hand, TME is a cellular community composed of tumor cells, endothelial cells, and stroma, which plays a key role in tumor control and progression, and is highly associated with malignant cell immune evasion, chemotherapy resistance, and tumor cell proliferation^64,65^. The expression of immunomodulators reflects the immunological characteristics of TME, MHC, receptors, chemokines, and immune stimulators are the main immunomodulators^66^. In the current study, we comprehensively analyzed the expression levels of immunomodulators between two groups. Interestingly, we discovered that most immunomodulators were highly expressed in the low-risk group, which contradicted our finding that the high-risk group would respond better to immunotherapy. Several findings demonstrated that the high expression of CCL11 and CCL19 predicts better OS of BRCA^67,68^, and Li et al.^69^ indicated that the high expression of CCL21 is highly associated with a lower distant recurrence rate of BRCA, through increasing the infiltration of CD8+T cells, thus, we confirmed that these studies may explain the up-regulation of immunomodulators in the low-risk group. Consequently, we hypothesized that the high-risk group would respond better to immunotherapy. In addition, we studied the abundances of the TIICs using CIBERSORT to evaluate the immune heterogeneity between high- and low-groups. Compared with the low-risk group, the increased tumor infiltrating abundances of macrophage M0, macrophage M2, neutrophil, and NK cell resting were discovered in the high-risk group. It has been recently highlighted that TIICs are a novel treatment target for immunotherapy of cancer^70^, in which macrophage M0 is a key factor in regulating immune responses^71^. Previous studies proved that the polarization of macrophage M2 is highly related to BRCA and that reducing macrophage M2 can help to suppress the process of BRCA^72,73^. These findings indicated that patients in the high-risk group have a worse prognosis, which may be related to the high abundance of macrophage M2. Collectively, the IL score revealed the vital role of TME in BRCA and might offer a novel strategy for the treatment of BRCA patients.

At present, immunotherapy is an approach to cancer treatment by activating the anti-tumor immune response, in which immune checkpoints are a group of inhibitory immune receptors that exert an immunosuppressive effect on the cell surface^74^, which can be beneficial to a subset of cancer patients in immunotherapy^75^. In this study, we found higher expression levels of immune checkpoints in the low-risk group, including PD-L1, TIM-3, VISTA, ADORA2A, BTLA, CD200, CD200R1, and KIR3DL1. Nevertheless, TIDE is shown to have better prognostic ability than other indicators^76^, we found a significantly lower TIDE score and T cell dysfunction score in the high-risk group, indicating a better response to immunotherapy in the high-risk group. In particular, PD-L1 inhibitor durvalumab and PARP inhibitor olaparib can be used for the treatment of HER2-negative BRCA^77^, our research found that the responders to durvalumab and olaparib therapy had higher risk score than non-responders in GSE173839 dataset, further suggesting that the BRAC patients in high-risk group response better to immunotherapy. Together, these results demonstrated that the IL score can be employed as a potential biomarker for BRCA immunotherapy. Docetaxel and paclitaxel are known to be commonly used chemotherapeutic drugs for BRCA^78,79^, and sensitivity analysis of chemotherapeutic drugs showed that the low-risk group had a significantly higher response to docetaxel and paclitaxel. Besides, neoadjuvant chemotherapy is a novel treatment method for BRCA, which aims to reduce the tumor size and improve OS in BRCA patients with locally advanced cancer^80^, and the combination neoadjuvant chemotherapy (paclitaxel + pertuzumab + trastuzumab) had a higher risk score in pCR. Consequently, we speculated that there is a potential interactive correlation between IL score and chemotherapeutic drug sensitivity of BRCA.

In summary, a 21-gene signature based on IL genes was developed and validated to have robust performance in predicting the survival outcomes of BRCA patients. In addition, the risk model was proved to provide novel insights into clinical characteristics, TME landscape, immunotherapy response, and chemotherapeutic drug sensitivity between high- and low-risk groups. Taken together, we believed that our findings could offer a powerful prognostic biomarker for individualized prediction of clinical decision-making and provide a theoretical basis for further studies in patients with BRCA.

## Supplementary Information

Supplementary Legends.

Supplementary Figure S1.

Supplementary Figure S2.

Supplementary Figure S3.

Supplementary Figure S4.

Supplementary Table S1.

Supplementary Table S2.

Supplementary Table S3.

Supplementary Table S4.

Supplementary Table S5.

Supplementary Table S6.

Supplementary Table S7.

## Data Availability

The datasets generated and analyzed during the current study are available in The Cancer Genome Atlas (TCGA) repository (http://xena.ucsc.edu/), and GSE22219, GSE25065, and GSE21653 from Gene Expression Omnibus (GEO) repository (https://www.ncbi.nlm.nih.gov/geo/). Access to both repositories is not required accession number. The datasets analyzed during the current study are available from the corresponding author on reasonable request.
