# Supplementary material for: Comprehensive evaluation of breast cancer immunotherapy and tumor microenvironment characterization based on interleukin genes-related risk model
Source: Sci Rep. 2022 Nov 28;12:20524. doi: 10.1038/s41598-022-25059-8 (PMC9705306; doi:10.1038/s41598-022-25059-8)
Supplement: Supplementary file 1 — Supplementary Legends. [file 41598_2022_25059_MOESM1_ESM.docx]

**Legends of supplementary tables**

**Supplementary table 1** There are 94 IL genes. IL: interleukins

**Supplementary table 2** 42 optimal IL genes screened by LASSO. LASSO: the least absolute shrinkage and selection operator

**Supplementary table 3** 21 intersection genes overlapped by RF and LASSO. RF: random forest,

**Supplementary table 4** The risk score of high- and low-risk groups in the TCGA cohort. TCGA: The Cancer Genome Atlas

**Supplementary table 5** The risk score of high- and low-risk groups in the GSE22219 cohort.

**Supplementary table 6** The risk score of high- and low-risk groups in the GSE25065 cohort.

**Supplementary table 7** The risk score of high- and low-risk groups in the GSE21653 cohort.
