# Supplementary figures and images for: Comprehensive evaluation of breast cancer immunotherapy and tumor microenvironment characterization based on interleukin genes-related risk model
Source: Sci Rep. 2022 Nov 28;12:20524. doi: 10.1038/s41598-022-25059-8 (PMC9705306; doi:10.1038/s41598-022-25059-8)

**
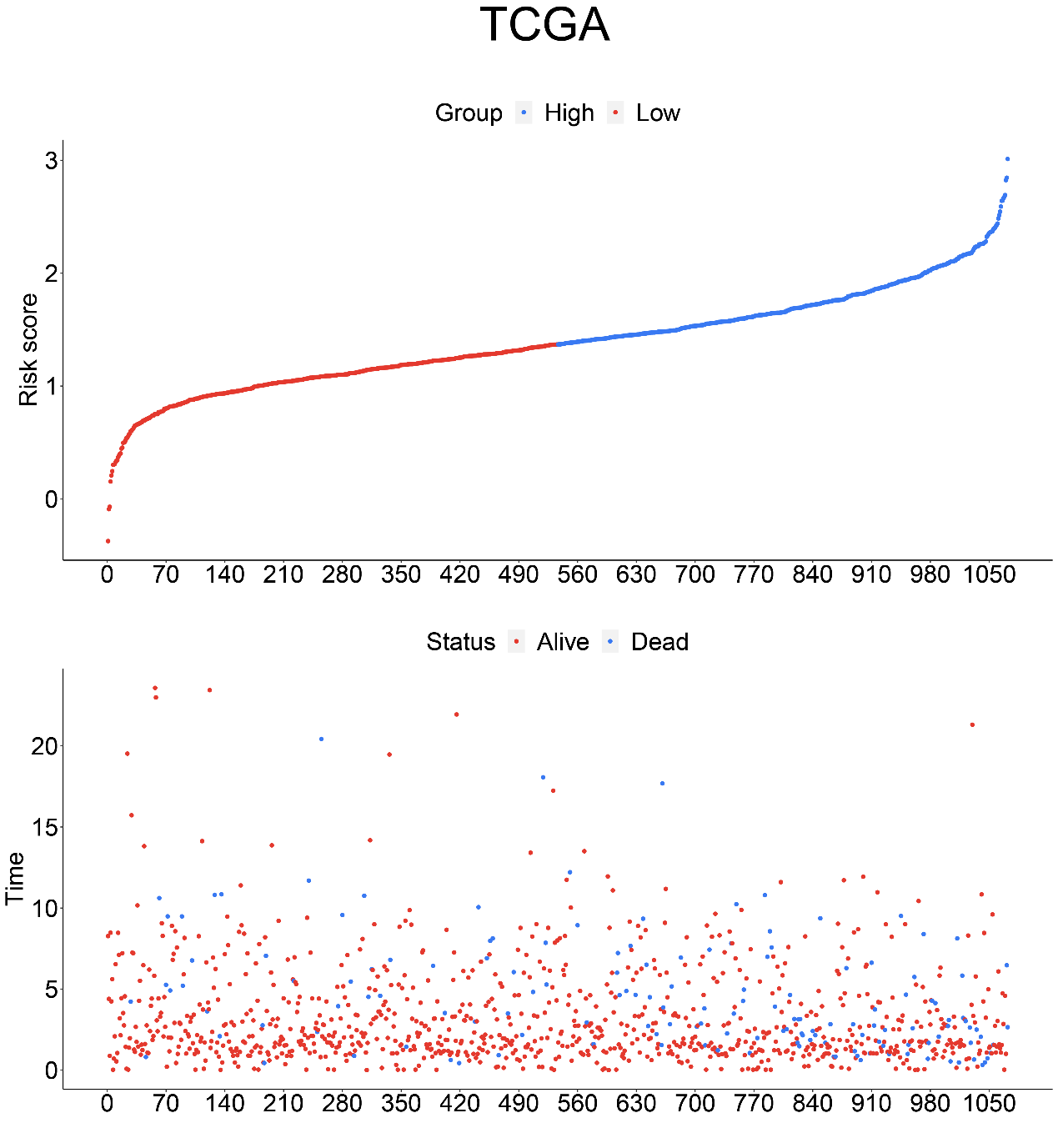
**

**Figure S1** Distribution of risk score and survival status for BRCA patients in the TCGA cohort.

Supplement: Supplementary file 2 — Supplementary Figure S1. [file 41598_2022_25059_MOESM2_ESM.docx]

**
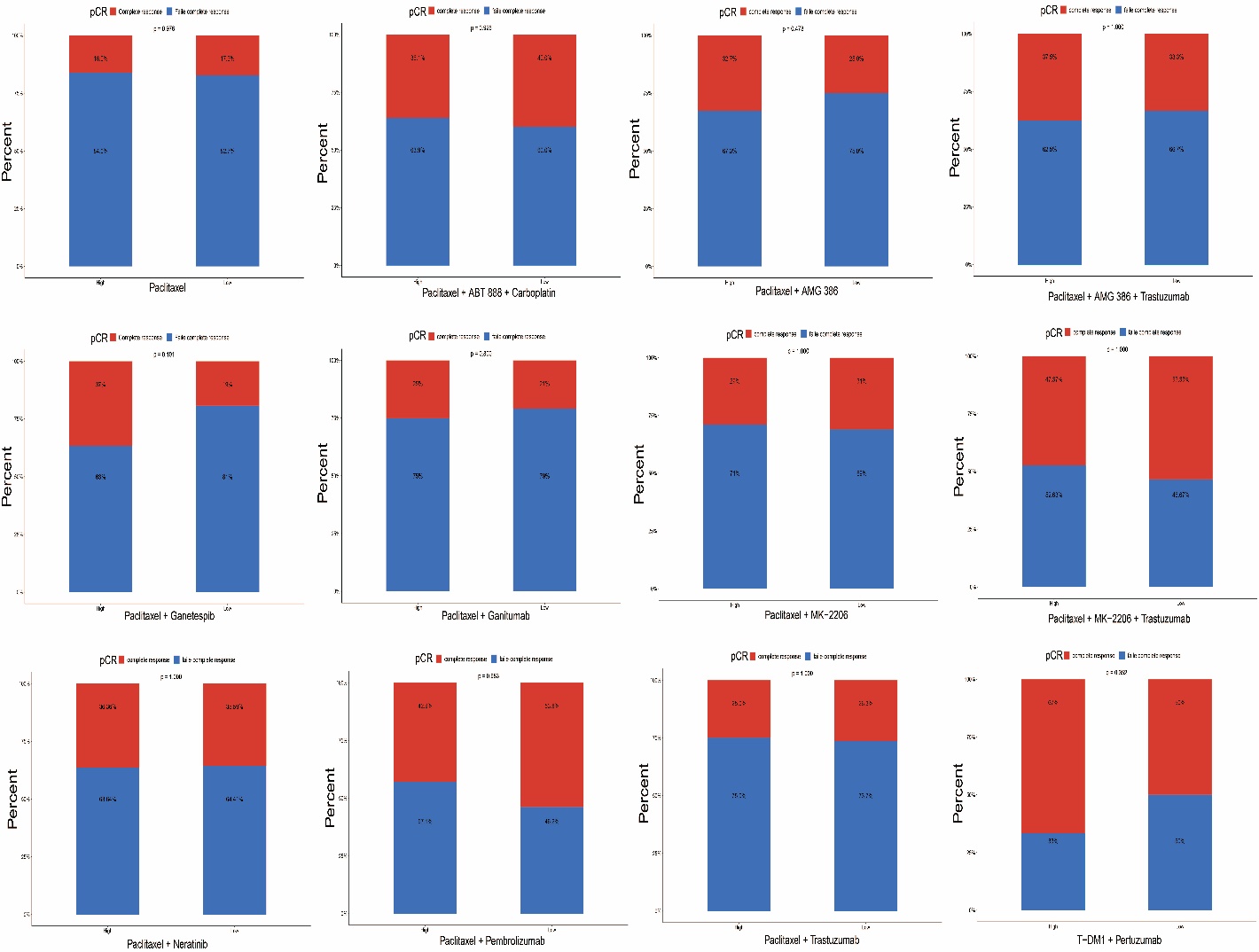
Figure S3** The pCR ratio of neoadjuvant chemotherapy in high- and low-risk groups.

Supplement: Supplementary file 4 — Supplementary Figure S3. [file 41598_2022_25059_MOESM4_ESM.docx]

**
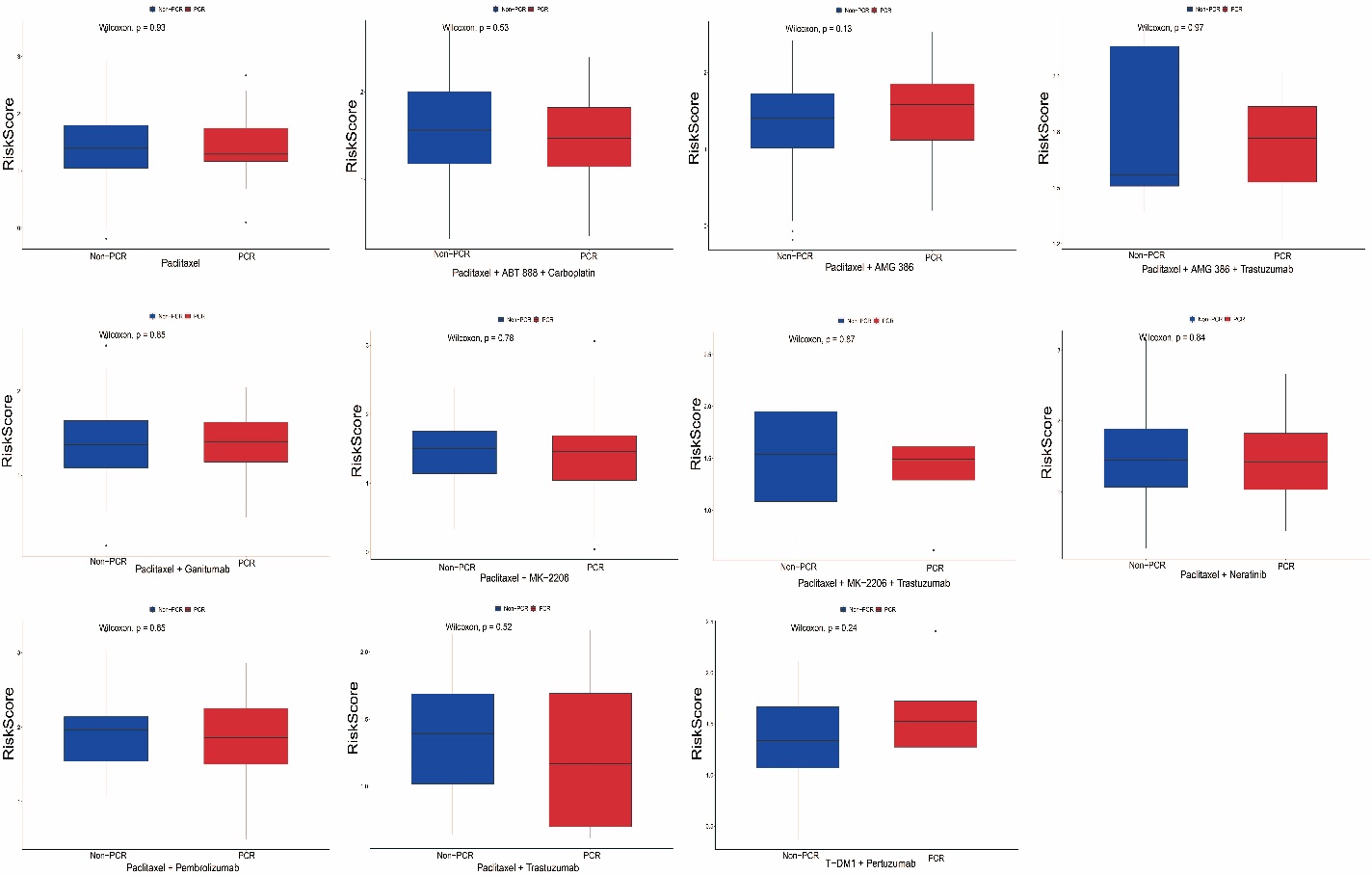
Figure S4** Risk scores for no-pCR and pCR with different neoadjuvant chemotherapy.

Supplement: Supplementary file 5 — Supplementary Figure S4. [file 41598_2022_25059_MOESM5_ESM.docx]
