# Supplementary material for: Comprehensive evaluation of breast cancer immunotherapy and tumor microenvironment characterization based on interleukin genes-related risk model
Source: Sci Rep. 2022 Nov 28;12:20524. doi: 10.1038/s41598-022-25059-8 (PMC9705306; doi:10.1038/s41598-022-25059-8)
Supplement: Supplementary file 3 — Supplementary Figure S2. [file 41598_2022_25059_MOESM3_ESM.docx]

**
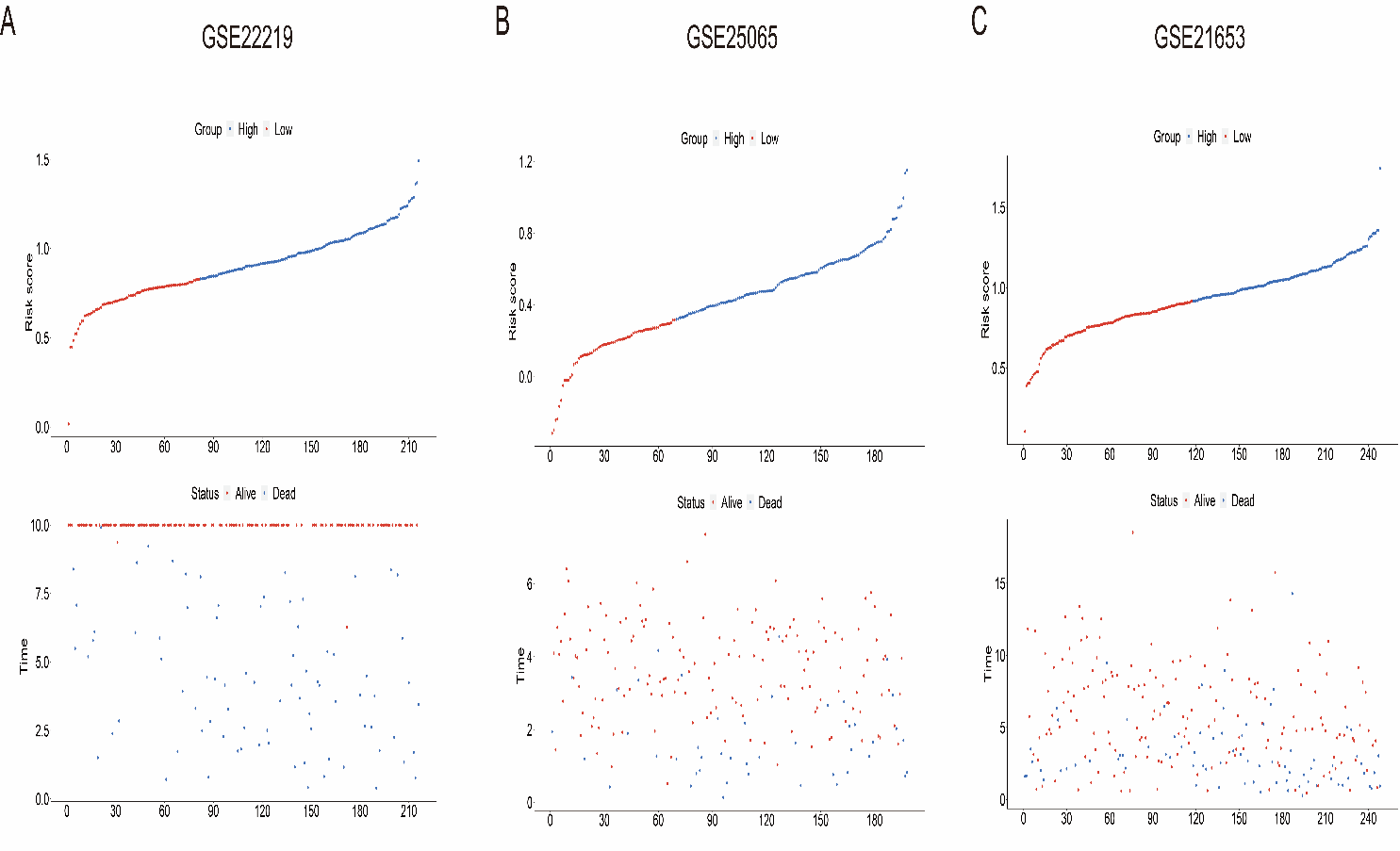
 Figure S2** Distribution of risk score and survival status for BRCA patients in GSE22219 cohort (A), GSE25065 cohort (B), and GSE21653 cohort (C).
